# Association of body fat and muscle tissue parameters with fatty liver disease identified by ultrasound

**DOI:** 10.1186/s12944-023-01933-w

**Published:** 2023-10-04

**Authors:** Xuan Song, Hongxia Wu, Bei Wang, Hongjun Sun

**Affiliations:** 1 Department of Medical Ultrasound, Shandong Provincial Qianfoshan Hospital and Health Key Laboratory of Abdominal Medical Imaging, The First Affiliated Hospital of Shandong First Medical University, Jinan, Shandong China; 2 Department of Nursing, The First Affiliated Hospital of Shandong First Medical University & Shandong Provincial Qianfoshan Hospital, Jinan, Shandong China

**Keywords:** Fatty liver disease, ultrasound, dual energy X-ray absorptiometry, body composition

## Abstract

**Aims:**

To examine the association between body fat and muscle parameters and FLD in individuals of Chinese descent.

**Methods:**

A total of 515 participants who underwent routine check-ups between November 2019 and August 2021 were reviewed. Based on ultrasound performance, the subjects were categorized into the non-FLD group and the FLD group. The prevalence of FLD in sex subgroups was analyzed using logistic regression to calculate the odds ratios (ORs) of body composition parameters with adjustment for confounders.

**Results:**

A total of 262 males and 253 females aged 20–84 years were reviewed. In both males and females, higher fat mass index (FMI) (OR: 1.989 for males vs. 1.389 for females), fat mass percent (FM%) (OR: 1.253 for males vs. 1.149 for females), visceral adipose tissue (VAT) (OR: 1.002 for males vs. 1.002 for females), and body mass index (BMI) (OR: 1.530 for males vs. 1.247 for females)were associated with increased ORs of FLD while higher lean mass percent (LM%) (OR: 0.839 for males vs. 0.856 for females)was associated with decreased ORs of FLD. Despite accounting for confounding factors, the associations remained present. Logistic regression of the quartiles of the indices showed associations with the prevalence of FLD. The trends still existed even after adjusting for confounders.

**Conclusion:**

Independently of age, lipid profiles and other confounders, lower VAT, FM, FMI, FM% and BMI tended to be associated with a lower prevalence of FLD, while lower LM% trended to be associated with a higher prevalence of FLD in both sexes of the general population.

## Introduction

Fatty liver disease (FLD), previously known as nonalcoholic fatty liver disease (NAFLD), is now defined as metabolic associated fatty liver disease (MAFLD), which specifically includes metabolic dysfunction and excludes factors such as excessive alcohol consumption and medication usage [1]. The accumulation of too much fat in the liver, known as FLD, can result in cirrhosis, hepatocellular carcinoma, or even fatality in severe instances [2]. FLD is believed to be a globally prevalent chronic liver disease, with its prevalence increasing in line with the growing prevalence of obesity [3]. A study demonstrated that the overall prevalence of FLD significantly increased from 25.5% (before 2005) to 37.8% (after 2016) [4].

Ultrasound is extensively utilized in clinical settings for the diagnosis of FLD due to its noninvasiveness, reproducibility, and ability to provide real-time imaging. By analyzing the echogenicity, contrast between the liver and kidney, and visibility of the hepatic vein, it is possible to identify and measure the extent of fat infiltration in the liver [5]. DXA, a widely accepted and accurate technique, is frequently employed to assess body composition, encompassing measurements of visceral adipose tissue (VAT), fat mass index (FMI), total fat mass (FM), fat mass percent (FM %), lean mass percent (LM%) and more [6]. The body composition parameters could describe the body fat and muscle distribution of an individual. Many studies have demonstrated that obesity is associated with FLD, independent of other metabolic factors, while other studies have concluded that a loss of muscle mass might also be associated with FLD [7–9]. Ramírez-Vélez et al found that liver fat content assessed by CAP was significantly correlated with higher FM, android FM, and VAT in youths with FLD [7]. In Ciardullo’s study, it was found that android fat deposition measured by DXA was associated with liver steatosis measured by controlled attenuation parameters in both sexes [8].

As the most common method to assess FLD, it has significant value to find the association between ultrasound-diagnosed FLD and body fat and muscle distribution parameters measured by DXA in clinic. However, there is a scarcity of studies examining the association between DXA-derived VAT, FM, FMI, FM% and LM% and the presence of FLD determined by ultrasound in Chinese individuals. In the present study, the association between several body composition parameters and FLD in the Chinese general population was estimated.

## Methods and subjects

This study is a retrospective analysis. A total of 515 individuals who received DXA scans and abdominal ultrasounds for routine check-ups at Shandong Qianfoshan Hospital between November 2019 and August 2021 were included. The research received approval from the ethics committee at Qianfoshan Hospital (No. S1181).

The exclusion criteria included individuals younger than 20; those who consumed more than 20 g of alcohol per day; individuals with autoimmune liver disease, viral hepatitis, or cirrhosis; individuals with other systemic diseases, severe chronic diseases, or malignant diseases; and individuals using medications that may disrupt fat infiltration.

### Laboratory tests and clinical data

Medical records were used to gather clinical information and blood examination results. Date on the patients’ age, heart rate, blood pressure, height, weight, alanine aminotransferase (ALT), aspartate aminotransferase (AST), γ-glutamyl transferase (GGT), fasting plasma glucose (FPG), triacylglycerol (TG), total cholesterol (Chol), high density lipoprotein (HDL) and low density lipoprotein (LDL) were collected from medical records. Hypertension was characterized by having a systolic blood pressure (SBP) equal to or greater than 140 mmHg, a diastolic blood pressure (DBP) equal to or greater than 90 mmHg, or the use of medication for hypertension [10]. Obesity was classified as having a body mass index (BMI) equal to or greater than 25 kg/m^2^ [11]. Diabetes mellitus (DM) was characterized by a fasting plasma glucose (FPG) level equal to or greater than 7.0 mmol/L or a prior diagnosis of diabetes [12]. After fasting overnight, blood samples were obtained from the median cubital vein, and the concentrations of ALT, AST, γ-GGT, TG, Chol, HDL, LDL, and FPG were measured.

### Ultrasound examination

The ultrasound database provided all the collected ultrasound images. All ultrasound examinations were executed by LOGIQ® E9 (GE 17.0). Three experienced ultrasound physicians with more than 5 years of experience reviewed the ultrasound images, and the inter- and inner ICC of ultrasound examination were analyzed in a previous study [5]. The subjects were categorized into normal, mild, moderate, and severe steatosis groups based on a previous study’s findings on ultrasound presentation, including liver echo, liver and kidney echo contrast, and the visibility of intrahepatic vessels and diaphragm [6]. The FLD group included individuals with mild, moderate, and severe steatosis. Hence, the entire population was categorized into two groups: the non-FLD group and the FLD group.

### DXA examination

The DXA database provided all the collected DXA data. A DXA scanner (GE, WI, USA) was used to measure whole-body scans, which were then analyzed automatically. Before the examination, every subject stayed still for a rest of 15 minutes. The subject is positioned supine on the examination table in a neutral position with the top of the head approximately 3 cm below the upper horizontal line of the scanning area, avoiding significant extension or flexion of the head. The upper extremities are positioned along the body with the palms of the hands down, and there should be at least approximately 1 cm of clearance between the upper extremities and the body. The feet should be neutral or slightly internally rotated, with space between the lower extremities. The ultimate accurate full body depiction should encompass the entirety of the patient’s body. DXA measurement parameters included body FM, fat mass percent (FM %), lean mass (LM), lean mass percent (LM %), upper/lower extremity FM, upper/lower extremity LM, and bone mineral content (BMC). Furthermore, additional calculations were performed, including the calculation of appendicular skeletal muscle (ASM) by combining upper extremity LM and lower extremity LM, and the evaluation of appendicular skeletal muscle mass index (ASMI) by dividing ASM by the square of height.

### Statistical analysis

The statistical analyses were performed using SPSS 24.0 software from IBM (Chicago, IL) and MedCalc. A P value below 0.05 was considered statistically significant. The normal distribution of the data was tested using the Kolmogorov‒Smirnov test. Percentages represent categorical variables, while medians ± interquartile ranges (IQRs) represent continuous variables. The chi-square test was employed to analyze categorical variables. The Mann‒Whitney U test was employed to compare the general information, blood biochemical parameters, and DXA parameters of the two groups. We performed logistic regression to determine the odds ratios (ORs) and 95% confidence intervals (95% CI) for VAT, BMI, LM%, and various other parameters related to body composition. We conducted multiple logistic regressions to control for confounding variables. Model 1 adjusted for age, while Model 2 adjusted for additional factors such as SBP, DBP, presence of hypertension (1 for yes, 0 for no), and presence of diabetes (1 for yes, 0 for no). Model 3 further adjusted for factors including ALT, AST, TG, GGT, Chol, HDL, LDL, and FPG.

## Results

1. Comparison of general information, blood biochemistry and body composition

A total of 515 subjects (262 males and 253 females) aged 20–84 years were included in the study. The clinical features and blood tests of the FLD and non-FLD groups and the sex subgroups are displayed in Tables 1 and 2. In females, the prevalence of type 2 diabetes (T2D) was higher in the FLD group than in the non-FLD group. There was no difference in the occurrence of T2D between the male FLD group and the male non-FLD group. Furthermore, there were no disparities detected in the occurrence of high blood pressure (HBP) between the FLD group and the non-FLD group in either sex subcategory. Both male and female subjects in the FLD group exhibited decreased HDL levels but increased weight, BMI, ALT, GGT, FPG, TGs, and TG/HDL compared to individuals in the non-FLD group (all P < 0.01). Moreover, age, height, AST, and Chol were significantly different between the non-FLD and FLD groups (all P < 0.05). According to DXA parameters, VAT, FM%, FMI, LMI, LM, and ASMI were significantly lower in the non-FLD group than in the FLD group in both sexes (P < 0.01), while LM% was significantly higher in the non-FLD group than in the FLD group (P < 0.001).

**Table 1:** The clinical characteristics and body compositions parameters of FLD group and non-FLD group in males

Parameters	Non-FLD group	FLD group	P values	
N	112	150		
Age	60.5(52.0, 67.8)	56.0(47.8, 62.0)	0.001	
HR	79.5(72.0, 86.0)	80.5(72.0, 90.0)	0.053	
SBP	134.0(121.0, 146.5)	134.5(121.0, 148.0)	0.431	
DBP	79.0(69.3, 86.8)	80.0(74.0, 92.0)	0.055	
HBP%	50(44.6%)	81(54.0%)	0.134	
T2D%	94(83.9%)	119(79.3%)	0.345	
Height(cm)	170.0(168, 175)	175.0(170, 178.3)	< 0.001	
Weight(kg)	67.6(62.7, 75.5)	82.9(75.7, 91.0)	< 0.001	
BMI(kg/m^2^)	23.6(21.7, 25.7)	27.6(25.3, 29.9)	< 0.001	
ALT	15.35(11.80, 20.43)	20.75(14.38, 30.98)	< 0.001	
AST	15.85(12.85, 20.38)	17.95(14.10, 22.93)	0.007	
FPG	6.47(4.98, 8.51)	7.28(5.74, 10.22)	0.003	
TG	1.03(0.72, 1.49)	1.65(1.16, 2.55)	< 0.001	
CHOL	4.15(3.43, 4.95)	4.46(3.78, 5.03)	0.04	
HDL	1.15(0.95, 1.37)	1.01(0.89, 1.16)	0.001	
LDL	2.29(1.79, 2.89)	2.50(2.00, 3.01)	0.056	
HbA1c	7.50(6.20, 9.10)	7.50(6.40, 9.18)	0.653	
GGT	20.0(13.85, 29.0)	27.6(19.5, 44.1)	< 0.001	
ALP	64.0(54.0, 82.8)	65.5(55.0, 81.3)	0.802	
TG/HDL	0.88(0.58, 1.48)	1.60(1.08, 2.64)	< 0.001	
VAT(g)	1156.0(781.0, 1542.5)	1966.5(1558, 2459.0)	< 0.001	
LM%	69.34(66.22, 73.69)	64.54(61.48, 67.42)	< 0.001	
LM(kg)	47.841(44.19, 52.41)	53.65(49.38, 59.00)	< 0.001	
FM%	27.45(23.03, 31.28)	32.95(29.90, 36.35)	< 0.001	
FM(kg)	18.73(14.41, 20.96)	26.19(22.00, 30.87)	< 0.001	
ASMI(kg/m^2^)	7.09(6.40, 7.58)	7.80(7.26, 8.54)	< 0.001	
LMI(kg/m^2^)	16.59(15.28, 17.44)	17.76(16.36, 18.79)	< 0.001	
FMI(kg/m^2^)	6.15(4.95, 7.20)	8.53(7.41, 10.56)	< 0.001	

*Abbreviation*: ALT: *alanine aminotransferase; ASMI appendicular skeletal muscle mass index, AST aspartate aminotransferase, BMI body mass index, DBP diastolic blood pressure, T2D type 2 diabetes mellitus, HBP high blood pressure, HR heart rate, GGT glutamyl transpeptadase, FM fat mass, FM% fat mass percentage, FMI fat mass index, FPG fasting plasma glucose, HDL high density lipoprotein, LDL low density lipoprotein, LM lean mass, LM% lean mass percentage, LMI lean mass index, SBP systolic blood pressure, TC total cholesterol, TG triacylglycerol, VAT visceral adipose tissue*

**Table 2:** The clinical characteristics and body compositions parameters of FLD group and non-FLD group in females

Parameters	Non-FLD group	FLD group	P values
N	104	149	
Age	60.0(52.0, 65.0)	59.0(52.5, 66.0)	0.980
HR	77.0(72.0, 84.0)	80.0(75.0, 88.5)	0.032
SBP	132.5(122.0, 145.5)	134.0(122.5, 149.0)	0.411
DBP	78.0(71.3, 85.8)	79.0(70.0, 86.0)	0.577
HBP%	34.0(33.3%)	69(45.7%)	0.050
T2D%	47(46.1%)	91(60.3%)	0.026
Height	160.0(155.0, 163.0)	160.0(158.0, 164.0)	0.071
Weight	58.6(52.8, 65.1)	65.6(59.8, 76.4)	< 0.001
BMI(kg/m^2^)	23.3(21.00, 25.5)	25.9(23.5, 29.6)	< 0.001
ALT	12.85(9.93, 18.38)	15.3(11.5, 23.65)	0.001
AST	17.4(14.03, 20.68)	16.5(14.0, 20.25)	0.624
FPG	5.11(4.47, 6.880	6.21(5.12, 9.08)	< 0.001
TG	1.14(0.82, 1.62)	1.38(0.98, 2.00)	0.002
CHOL	4.65(3.62, 5.45)	4.82(3.99, 5.61)	0.300
HDL	1.30(1.08, 1.51)	1.13(0.98, 1.37)	0.001
LDL	2.80(1.80, 3.27)	2.90(2.28, 3.41)	0.088
HbA1c	6.40(5.60, 8.60)	6.80(5.95, 8.85)	0.051
GGT	16.6(12.00, 24.7)	20.0(15.0, 28.0)	0.003
ALP	71.0(57.0, 90.8)	72.0(60.0, 87.5)	0.666
TG/HDL	0.87(0.62, 1.40)	1.14(0.78, 1.77)	0.001
VAT(g)	746.0(521.0, 953.8)	1227.0(963.5, 1582.5)	< 0.001
LM%	61.1(58.4, 64.1)	56.9(53.5, 60.3)	< 0.001
LM(kg)	35.91(33.00, 39.51)	39.15(35.19, 42.74)	< 0.001
FM%	36.80(33.45, 39.60)	41.30(37.90, 45.40)	< 0.001
FM(kg)	20.19(17.97, 24.25)	26.19(22.53, 32.45)	< 0.001
ASMI(kg/m^2^)	5.93(5.37, 6.47)	6.27(5.74, 6.94)	0.001
FMI(kg/m^2^)	7.94(6.75, 9.58)	10.27(8.70, 12.58)	< 0.001
LMI(kg/m^2^)	14.05(12.95, 15.37)	14.96(13.83, 16.25)	< 0.001

*Abbreviation*: ALT: *alanine aminotransferase; ASMI appendicular skeletal muscle mass index, AST aspartate aminotransferase, BMI body mass index, DBP diastolic blood pressure, T2D type 2 diabetes mellitus, HR heart rate, GGT glutamyl transpeptadase, FM fat mass, FM% fat mass percentage, FMI fat mass index, FPG fasting plasma glucose, HDL high density lipoprotein, LDL low density lipoprotein, LM lean mass, LM% lean mass percentage, LMI lean mass index, SBP systolic blood pressure, TC total cholesterol, TG triacylglycerol, VAT visceral adipose tissue*

2. Correlation between adiposity indices and muscle mass parameters and FLD

The association between body composition and FLD was examined in both male and female subcategories. First, logistic regressions were performed on the continuous variables of BMI, VAT, FM%, FMI, and LM%. The findings indicated a positive association between the presence of FLD and elevated BMI, VAT, FM%, and FMI in both male and female subgroups. These associations remained significant even after controlling for confounding factors in Model 1, Model 2, and Model 3. With an incremental rise in the above parameters, both male and female subcategories demonstrated a heightened susceptibility to FLD. In males, the ORs (95% CI) of BMI, VAT, FM%, and FMI in Model 3 were OR: 1.530, 95% CI: 1.362, 1.719; OR: 1.002, 95% CI: 1.001, 1.002; OR: 1.253, 95% CI: 1.174, 1.337; and OR: 1.989, 95% CI: 1.644, 2.407, respectively. In females, the ORs (95% CI) of BMI, VAT, FM%, and FMI in Model 3 were OR: 1.247, 95% CI: 1.153, 1.349; OR: 1.002, 95% CI: 1.002, 1.003; OR: 1.149, 95% CI: 1.093, 1.208; and OR: 1.389, 95% CI: 1.238, 1.557, respectively. The presence of FLD showed a negative correlation with LM%. As the LM% increased, the presence of FLD tended to decrease. The ORs were 0.839 (95% CI: 0.792, 0.888) in males and 0.856(95% CI: 0.811, 0.904) in females in Model 3 (Tables 3 and 4).

**Table 3:** The association of BMI、VAT、FM%、FMI and LM% as continuous variables with FLD in logistic regression models in males

Parameters	Unadjusted OR(95%CI)	P	Model 1 OR(95%CI)	P	Model 2 OR(95%CI)	P	Model 3 P OR(95%CI)
BMI	1.530(1.362, 1.719)	< 0.001	1.530(1.362, 1.719)	< 0.001	1.530(1.362, 1.719)	< 0.001	1.483(1.313, 1.677) <0.001
VAT	1.002(1.001, 1.002)	< 0.001	1.002(1.001, 1.003)	< 0.001	1.002(1.001, 1.003)	< 0.001	1.002(1.001, 1.002) <0.001
FM%	1.253(1.174, 1.337)	< 0.001	1.272(1.187, 1.364)	< 0.001	1.253(1.174, 1.337)	< 0.001	1.250(1.161, 1.347) <0.001
FMI	1.989(1.644, 2.407)	< 0.001	2.063(1.688, 2.521)	< 0.001	1.989(1.644, 2.407)	< 0.001	1.924(1.562, 2.369) <0.001
LM%	0.839(0.792, 0.888)	< 0.001	0.955(0.930, 0.980)	< 0.001	0.831(0.782, 0.883)	< 0.001	0.855(0.801, 0.912) <0.001

*Model 1: adjusted for age, Model 2: adjusted for model 1 + SBP, DBP, presence of hypertension, and presence of diabetes, Model 3: adjusted for model 1 + model 2 + ALT, AST, TG, GGT, Chol, HDL, LDL, and FPG.*

*Abbreviation: BMI body mass index, CI confidence interval, FMI fat mass index, FM% fat mass percentage, LM% lean mass percentage, OR odds ratio, VAT visceral adipose tissue. Model 1 adjusted for age, Model 2 adjusted for model 1 + SBP, DBP, presence of hypertension, and presence of diabetes, Model 3 adjusted for model 1 + model 2 + ALT, AST, TG, GGT, Chol, HDL, LDL, and FPG.*

**Table 4:** The association of BMI、VAT、FM%、FMI and LM% as continuous variables with FLD in logistic regression models in females

Parameters	Unadjusted OR(95% CI)	P	Model 1 OR(95% CI)	P	Model 2 OR(95% CI)	P	Model 3 OR(95% CI)	P
BMI	1.247(1.153, 1.349)	< 0.001	1.250(1.156, 1.352)	< 0.001	1.250(1.156, 1.352)	< 0.001	1.253(1.146, 1.369)	< 0.001
VAT	1.002(1.002, 1.003)	< 0.001	1.002(1.002, 1.003)	< 0.001	1.002(1.002, 1.003)	< 0.001	1.002(1.002, 1.003)	< 0.001
FM%	1.149(1.093, 1.208)	< 0.001	1.178(1.116, 1.245)	< 0.001	1.178(1.116, 1.245)	< 0.001	1.153(1.089, 1.221)	< 0.001
FMI	1.389(1.238, 1.557)	< 0.001	1.426(1.267, 1.606)	< 0.001	1.426(1.267, 1.606)	< 0.001	1.377(1.214, 1.563)	< 0.001
LM%	0.856(0.811, 0.904)	< 0.001	0.856(0.811, 0.904)	< 0.001	0.835(0.787, 0.886)	< 0.001	0.852(0.801, 0.907)	< 0.001

*Model 1: adjusted for age, Model 2: adjusted for model 1 + SBP, DBP, presence of hypertension, and presence of diabetes, Model 3: adjusted for model 1 + model 2 + ALT, AST, TG, GGT, Chol, HDL, LDL, and FPG.*

*Abbreviation: BMI body mass index, CI confidence interval, FMI fat mass index, FM% fat mass percentage, LM% lean mass percentage, OR odds ratio, VAT visceral adipose tissue. Model 1 adjusted for age, Model 2 adjusted for model 1 + SBP, DBP, presence of hypertension, and presence of diabetes, Model 3 adjusted for model 1 + model 2 + ALT, AST, TG, GGT, Chol, HDL, LDL, and FPG.*

3. Then, logistic regression based on quartiles of BMI, VAT, FM%, FMI and LM% in the Q1, Q2, Q3, and Q4 groups was performed and wans adjusted for confounders. With increasing BMI, VAT, FM%, and FMI quartiles in the Q2-Q4 groups, the corresponding ORs also increased gradually compared to the Q1 groups. However, with an increase in LM% quartiles in the Q2-Q4 groups, the ORs decreased gradually compared to the Q1 groups. The associations remained intact despite accounting for confounding variables in Model 1, Model 2, and Model 3. In Model 3, when comparing the OR in the Q1 group with the adjusted ORs in the Q2-Q4 group for adiposity indices, the OR range for males was 2.339–29.796 and for females was 2.049–37.721 (all P < 0.001) (Tables 5 and 6).

**Table 5:** The association of BMI、VAT、FM%、FMI and LM% as categorical variables with FLD in logistic regression models in males

Parameters	Unadjusted OR(95% CI)	P		Model 1 OR(95% CI)	P	Model 2 OR(95% CI)	P	Model 3 OR(95% CI)	P
BMI									
Q1	1		1			1		1	
Q2	6.32(2.50, 16.01)	< 0.001	6.72(2.62, 17.26)		< 0.001	6.72(2.62, 17.26)	< 0.001	7.66(2.761, 21.24)	< 0.001
Q3	12.91(5.12, 32.55)	< 0.001	13.68(5.33, 35.08)		< 0.001	13.68(5.33, 35.08)	< 0.001	12.22(4.44, 33.66)	< 0.001
Q4	81.91(24.45, 274.44)	< 0.001	74.48(22.02, 251.94)		< 0.001	74.48(22.02, 251.94)	< 0.001	66.00(18.52, 235.22)	< 0.001
VAT									
Q1	1		1			1		1	
Q2	2.64(0.86, 8.14)	0.009	3.58(1.10, 11.65)		0.034	3.58(1.10, 11.65)	0.034	4.42(2.08, 9.41)	0.047
Q3	11.73(4.10, 33.60)	< 0.001	16.63(5.42, 51.03)		< 0.001	16.63(5.42, 51.03)	< 0.001	19.70(7.08, 54.78)	< 0.001
Q4	32.22(10.99, 94.47)	< 0.001	54.35(16.62, 177.71)		< 0.001	54.35(16.62, 177.71)	< 0.001	23.02(4.85, 109.29)	< 0.001
FM%									
Q1	1		1			1		1	
Q2	4.76(2.64, 8.57)	< 0.001	5.94(3.17, 11.13)		< 0.001	5.94(3.17, 11.13)	< 0.001	4.70(2.39, 9.23)	< 0.001
Q3	23.10(6.60, 80.80)	< 0.001	31.53(8.50, 116.98)		< 0.001	31.53(8.50, 116.98)	< 0.001	25.08(6.47, 92.22)	< 0.001
Q4	21.24(4.68, 96.24)	< 0.001	16.59(3.35, 82.16)		0.001	16.59(3.35, 82.16)	< 0.001	13.24(2.50, 70.17)	0.001
FMI									
Q1	1		1			1		1	
Q2	7.59(3.82, 15.09)	< 0.001	8.90(4.35, 18.25)		< 0.001	8.90(4.35, 18.25)	< 0.001	8.19(3.74, 17.91)	< 0.001
Q3	14.24(6.61, 33.40)	< 0.001	17.23(7.039, 42.17)		< 0.001	17.23(7.04, 42.17)	< 0.001	13.14(5.08, 33.99)	< 0.001
Q4	35.59(11.36, 111.47)	< 0.001	33.55(10.24, 109.92)		< 0.001	33.5(10.24, 109.92)	< 0.001	27.68(7.91, 96.88)	< 0.001
LM%									
Q1	1		1			1		1	
Q2	19.32(4.24, 88.04)	< 0.001	17.32(5.85, 79.94)		0.001	17.14(2.99, 73.97)	0.001	12.47(2.35, 66.20)	0.003
Q3	13.31(4.76, 37.23)	< 0.001	16.07(3.23, 51.36)		< 0.001	14.88(2.99, 73.97)	< 0.001	11.59(3.57, 37.52)	< 0.001
Q4	4.50(2.50, 8.07)	< 0.001	5.46(2.94, 10.15)		< 0.001	5.19(2.75, 9.81)	< 0.001	3.82(1.91, 7.65)	< 0.001

*Model 1: adjusted for age, Model 2: adjusted for model 1 + SBP, DBP, presence of hypertension, and presence of diabetes, Model 3: adjusted for model 1 + model 2 + ALT, AST, TG, GGT, Chol, HDL, LDL, and FPG.*

*Abbreviation: ASMI appendicular skeletal muscle mass index, BMI body mass index, CI confidence interval, FLD fat liver disease, FMI fat mass index, FM% fat mass percentage, LM% lean mass percentage, OR odds ratio, VAT visceral adipose tissue.*

**Table 6:** The association of BMI、VAT、FM%、FMI and LM% as categorical variables with FLD in logistic regression models in females

Parameters	Unadjusted (OR, 95% CI)	P	Model 1 (OR, 95% CI)	P	Model 2 (OR, 95% CI)	P	Model 3 (OR, 95% CI)	P
BMI								
Q1	1		1		1		1	
Q2	2.64(1.33, 5.23)	0.006	2.46(1.23, 4.94)	0.006	2.46(1.23, 4.94)	0.011	2.66(1.17, 6.04)	0.02
Q3	4.57(2.15, 9.69)	< 0.001	4.22(1.97, 9.03)	< 0.001	4.22(1.97, 9.03)	< 0.001	4.19(1.74, 10.10)	0.001
Q4	8.39(3.74, 18.84)	< 0.001	8.86(3.90, 20.15)	< 0.001	8.86(3.90, 20.15)	< 0.001	6.81(2.71, 17.15)	< 0.001
VAT								
Q1	1		1		1		1	
Q2	3.563(1.89, 6.70)	< 0.001	3.61(1.91, 6.81)	< 0.001	3.56(1.89, 6.70)	< 0.001	4.42(2.08, 9.42)	< 0.001
Q3	14.355(5.79,35.60)	< 0.001	14.59(5.86, 36.31)	< 0.001	14.36(5.79, 35.60)	< 0.001	19.76(7.10, 54.94)	< 0.001
Q4	17.816(4.97, 63.83)	< 0.001	17.940(5.00, 64.36)	< 0.001	17.82(4.97, 63.83)	< 0.001	10.18(3.01,34.42)	< 0.001
FM%								
Q1	1		1		1		1	
Q2	2.49(0.76, 8.16)	0.013	2.49(0.76, 8.16)	0.013	2.27(0.67, 7.69)	0.018	2.05(0.55, 7.67)	0.028
Q3	2.21(0.80, 6.14)	0.012	2.21(0.80, 6.14)	0.012	2.37(0.83, 6.76)	0.011	1.94(0.62, 6.06)	0.026
Q4	11.33(3.94, 32.62)	< 0.001	11.33(3.94, 32.62)	< 0.001	15.52(5.10, 47.29)	< 0.001	11.53(3.48, 38.18)	< 0.001
FMI								
Q1	1		1		1		1	
Q2	1.31(0.53, 3.24)	0.046	1.31(0.53, 3.24)	0.038	1.31(0.52, 3.30)	0.046	1.13(0.39, 3.25)	0.008
Q3	4.00(1.72, 9.29)	0.001	4.00(1.72, 9.29)	0.001	4.44(1.86, 10.58)	0.01	3.55(1.31, 9.63)	0.013
Q4	9.09(3.75, 22.05)	< 0.001	9.09(3.75, 22.05)	< 0.001	11.04(4.39, 27.78)	< 0.001	7.89(2.82, 22.09)	< 0.001
LM%								
Q1	1		1		1		1	
Q2	12.49(4.32, 36.09)	< 0.001	12.88(4.43, 37.49)	< 0.001	16.82(5.41, 52.30)	< 0.001	10.22(3.00, 34.82)	< 0.001
Q3	2.86(0.87, 9.37)	0.017	2.97(0.90, 9.81)	0.016	2.68(0.78, 9.17)	0.018	2.00(0.52, 7.72)	0.048
Q4	2.06(0.74, 5.74)	0.038	2.09(0.75, 5.83)	0.047	2.08(0.72, 5.98)	0.012	1.39(0.43, 4.43)	0.032

*Model 1: adjusted for age, Model 2: adjusted for model 1 + SBP, DBP, presence of hypertension, and presence of diabetes, Model 3: adjusted for model 1 + model 2 + ALT, AST, TG, GGT, Chol, HDL, LDL, and FPG.*

*Abbreviation: ASMI appendicular skeletal muscle mass index, BMI body mass index, CI confidence interval, FLD fat liver disease, FMI fat mass index, FM% fat mass percentage, LM% lean mass percentage, OR odds ratio, VAT visceral adipose tissue.*

## Discussion

This study evaluated the association between various body composition parameters measured by DXA and the diagnosis of FLD using ultrasound. According to a prior investigation, there were notable disparities in blood biochemical parameters and body composition parameters when comparing males and females [13–15]. Thus, this study compared the disparities in sex subgroups between the non-FLD group and FLD group. The study yielded valuable findings regarding the association between ultrasound-detected FLD and BMI and DXA-measured VAT, FM, FMI, LM%, and FM% variables. The results support the idea that a growing prevalence of FLD is linked to an upward trajectory of obesity. The positive association between FLD and BMI, VAT, FMI, LM%, and FM% is consistent with previous research [7, 8].

BMI is the most commonly used alternative indicator of body fat content. However, BMI does not reflect the content and distribution of body fat because BMI may not differentiate muscle mass from fat mass, suggesting that a high BMI may not necessarily indicate true obesity [16]. A significant association between the prevalence of FLD and BMI has been demonstrated. According to a study, it was found that a greater BMI was linked to a higher occurrence of FLD in Japan [16]. Conversely, a lower BMI was associated with a reduced incidence of FLD [17, 18]. In the study conducted by Chang et al. [19], it was found that the adjusted likelihood of developing FLD was higher in obese populations than in individuals with normal weight. The likelihood further increased after adjusting for various confounding factors. Furthermore, these associations persisted when the data were analyzed separately for different age groups and sexes. The findings were in line with the outcomes in the current investigation. This could be because individuals with higher BMI often have higher levels of body fat and increased free fatty acids (FFAs) in the liver through the portal effect. Furthermore, individuals with elevated BMI also tend to have a high dietary fat intake, consequently resulting in an elevation of FFAs within the liver.

VAT is an indicator of central adiposity. Numerous studies have discovered that the buildup of VAT is associated with a higher likelihood of developing metabolic disorders and cardiovascular ailments [20–22]. Moreover, it holds considerable clinical significance in relation to FLD [23]. In patients with type 2 diabetes mellitus (T2DM), VAT was associated with the severity of hepatic steatosis and liver stiffness, independent of BMI [20]. In the study, Busetto et al. [24] discovered that the sole factor linked to FLD in severely overweight women was the volume of VAT. According to a study conducted by KO et al. [25], individuals with FLD, both males and females, exhibited a greater amount of visceral fat area (VFA) than the general population. VAT tissue, as well as dietary sources could derive FFAs, which could be released to the portal venous system. Excess FFAs and chronic low-grade inflammation caused by VAT play important roles in liver injury progression in FLD [23].

FM, FM% and FMI were utilized as measures of overall body fatness and were also associated with an increased prevalence of FLD. Excessive consumption of fat or increased breakdown of fat leads to an abundance of circulating potent FFAs. The accumulation of triglycerides in both the liver and skeletal muscle subsequently leads to insulin resistance (IR) [26]. In this study, the FLD group had higher BMI, VAT, FMI, and FM% than the non-FLD group in both the male and female groups. The reason for this could be that the participants in the research were from the general public, where individuals with higher BMI tend to have higher amounts of VAT, FM, and FM%. This is in contrast to athletes, in whom BMI was not shown to be strongly correlated with VAT due to their lower proportion of FM compared to the general population. Patients experiencing malnutrition typically exhibit a low BMI, whereas patients with obesity and overnutrition often display a high BMI due to an elevation in visceral fat.

Additional research has found that the reduction in muscle mass is a novel contributing factor to FLD [27, 28]. To evaluate muscle mass, one can utilize LM, LMI, and LM%. When VAT increases, the reduction in lipocalin secretion leads to decreased muscle cell metabolism and increased lipid uptake [29]. Because insulin-mediated glucose uptake and utilization can involve skeletal muscle, muscle loss could potentially result in IR. According to the research conducted by Koo and colleagues (2014) [30], there is a correlation between the reduction in muscle mass and the presence of hepatic steatosis and hepatic fibrosis, regardless of IR and obesity. A comprehensive study with a significant number of participants investigated the impact of a relatively higher LM on the deceleration of FLD progression or the reversal of preexisting FLD [31]. The study revealed that the FLD group had a higher FMI than the non-FLD group, whereas the LM% in the FLD group was significantly lower than that in the non-FLD group.

Ultrasound is widely used as a noninvasive diagnostic tool for assessing FLD. The correlation between ultrasound-diagnosed FLD and different measures of adiposity and muscle, including VAT, FMI, FM%, LM, and LM%, has been increasingly studied. The research exhibited a notable association between the existence of FLD as identified through ultrasound and measures of body fatness. The promotion of insulin resistance (IR) and inflammation, which contributes to the development and progression of FLD, is believed to be a result of the increased deposition of VAT. Moreover, an increased FMI and FM% have additionally been discovered to have a positive correlation with the occurrence of FLD, suggesting the involvement of overall body fat accumulation in the condition. Identifying the risks for FLD shows the potential significance of these correlations in a clinical context. Typically, higher BMI, FMI, FM%, VAT and lower LM% measurements can offer healthcare professionals valuable insights into an individual’s adiposity profile, aiding in the identification of patients who could benefit from early intervention to prevent or manage FLD.

Furthermore, it is necessary to conduct more research to clarify the fundamental processes connecting FLD and physical constitution, as well as to examine the significance of these factors in forecasting the effectiveness of treatment and the advancement of diseases. Additionally, these correlations have implications for monitoring treatment efficacy and prognosis. BMI, VAT, FMI, FM% and LM% can be used to track changes in body fat distribution and assess the response to lifestyle modifications or pharmacological interventions. A reduction in these adiposity indices and an increase in LM% may indicate improvements in liver health and metabolic profiles.

### Strengths and weaknesses

The association between FLD identified through ultrasound and variables related to body composition has important consequences for both clinical practice and research. The study has multiple advantages. First, the study included separate analyses for male and female subgroups and compared the parameters related to body composition between the normal and FLD groups. Second, BMI, VAT, FM FMI and LM% were analyzed using logistic regression as continuous and categorical variables to calculate odds ratios. By utilizing both types of variables in our study, a more comprehensive and robust analysis could be obtained. The study demonstrated the association between ultrasound-diagnosed FLD and several common body compositions, which could provide more information on patient nutrition for the clinic.

Additionally, there are a few drawbacks in this research. In this study, the sample size was small and from a single center. Furthermore, this retrospective analysis was unable to establish a cause-and-effect connection between FLD and body composition. It is necessary to rectify these issues in future studies.

## Conclusion

In summary, the associations of ultrasound-detected FLD with BMI, VAT, FMI, FM%, and LM% are clinically meaningful in various aspects. The measures of obesity indices and muscle mass were found to have a positive association with the prevalence of FLD, whereas the LM%, compared to total muscle mass and body fatness, showed a negative association with FLD. In the general population, lower VAT, FM, FMI, FM% and BMI tended to be associated with a lower prevalence of FLD, while higher LM% trended to be associated with a high prevalence of FLD in both sexes of the general population. These results aid in identifying individuals at risk and monitoring treatment efficacy. This study provides information for enhancing management and preventing strategies in FLD in routine clinical practice.

## Data Availability

The datasets analyzed during the current study available from the corresponding author on reasonable request.
